# Aggressive lung involvement in a patient with T‐acute lymphoblastic leukaemia/lymphoblastic lymphoma: a tricky and rare case report

**DOI:** 10.1002/rcr2.614

**Published:** 2020-07-02

**Authors:** Chika Miyaoka, Takeshi Saraya, Kojiro Honda, Masachika Fujiwara, Haruyuki Ishii, Hajime Takizawa

**Affiliations:** ^1^ Department of Respiratory Medicine Kyorin University Tokyo Japan; ^2^ Department of Pathology Kyorin University Tokyo Japan

**Keywords:** Lung involvement, T‐acute lymphoblastic leukaemia/lymphoblastic lymphoma, thoracic computed tomography

## Abstract

A 39‐year‐old man was admitted to our university hospital because of diffuse pulmonary infiltrates on chest X‐ray. He had been diagnosed with T‐acute lymphoblastic leukaemia/lymphoblastic lymphoma three years before and had been treated with chemotherapy and cord blood stem cell transplantation twice. Although he had neither blast cells in the peripheral blood nor leucocytosis, urgent bronchoscopy findings demonstrated blast cells invading both the alveolar spaces/alveolar septa and the vein walls. These pathological findings corresponded to ground‐glass opacities and thickening of the interlobular septa on thoracic computed tomography (CT). In acute lymphoblastic leukaemia/lymphoblastic lymphoma patients presenting with infiltrates on thoracic CT, leukaemic pulmonary involvement should be considered in the differential diagnoses, even in the absence of hyperleucocytosis or blast cells in the blood, similar to pulmonary involvement in myeloid leukaemias.

## Graphical abstract

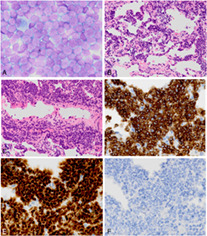

## Introduction

Acute lymphoblastic leukaemia/lymphoblastic lymphoma (ALL/LBL) is a clonal haematopoietic stem cell disorder of B‐ or T‐cell origin and accounts for 2–5% of adult non‐Hodgkin lymphoma, while lung involvement is scarcely reported. If the total number of blast cells in the peripheral blood is markedly elevated, myeloid leukaemias can invade the lung and presents as bilateral thickening of broncho‐vascular bundles (BVBs), interlobular septa, nodules, and consolidation on thoracic computed tomography (CT) [1]. In contrast, radiological and pathological correlation regarding T‐acute lymphoblastic leukaemia/lymphoblastic lymphoma (T‐ALL/LBL) has been scarcely reported so far. Herein, we described an extremely rare case of T‐ALL/LBL accompanied by massive lung involvement without lymphoblasts in the blood.

## Case Report

A 39‐year‐old man was admitted to our university hospital because of massive lung infiltration noted on chest radiography. He was diagnosed with T‐ALL/LBL three years before and had been treated with induction chemotherapy based on the Japan Adult Leukaemia Study Group ALL202 (JALSG ALL202) guidelines (cyclophosphamide 1200 mg/m^2^, daunomycin 60 mg/m^2^, vincristine 1.3 mg/m^2^, L‐asparaginase 3000 U/m^2^, and prednisolone 60 mg/m^2^).The patient had also undergone cord blood stem cell transplantation twice. He developed a left subcutaneous abdominal mass that had appeared two months previously.

**Figure 1:**
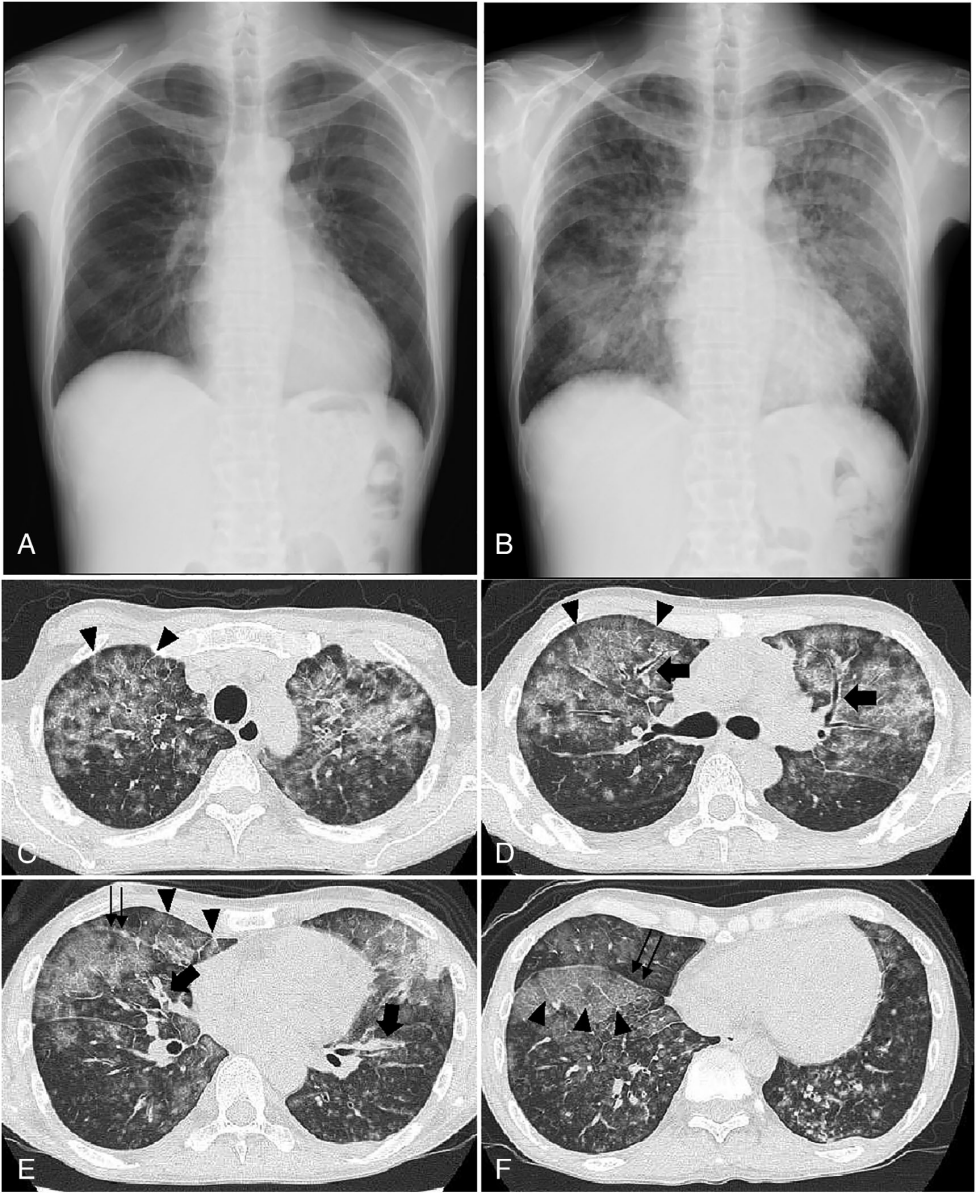
Normal chest X‐ray findings one month before admission to our hospital (A). Diffuse pulmonary infiltrates was noted in both lung fields at the time of admission (B). Non‐enhanced thoracic computed tomography (CT) on admission demonstrated ground‐glass opacity in both the lungs (C–F) with thickening of broncho‐vascular bundles (D, E; arrows), interlobular septa (C–F; arrow heads) and right interlobar fissure (D, E; double arrow). Randomly distributed tiny nodules were predominantly seen in both the lower lungs (E, F).

The vital signs and physical findings were normal except for subcutaneous abdominal mass at the lower left side of his abdomen. No adventitious sounds were recognized. Chest radiographs taken two months (figure not shown) and one month before (Fig. 1A) the patient's admission to our hospital reported no abnormal findings; however, an X‐ray taken at the time of admission showed a widespread infiltrate within both the lungs (Fig. 1B). Non‐enhanced thoracic CT demonstrated abundant pan‐lobular ground‐glass opacities (GGOs) in both the lungs (Fig. 1C–F) together with thickening of BVBs (Fig. 1D, E; arrows), interlobular septa (Fig. 1C–F; arrowheads), and right interlobar fissure (Fig. 1D, E; double arrow). Scattered tiny nodules were predominantly seen in the lower region of both the lungs (Fig. 1E, F). No apparent mediastinal lymphadenopathies were noted. Serum laboratory data showed normal white blood cell count (5100/μL) and no blast cells. Serum C‐reactive protein level was almost normal (0.71 mg/dL), while alkaline phosphatase (626 IU/L) and lactate dehydrogenase (395 IU/L) levels were moderately elevated. The results of serum β‐D‐glucan, galactomannan antigen, cryptococcus antigen, and interferon‐gamma release assays were all negative.

**Figure 2:**
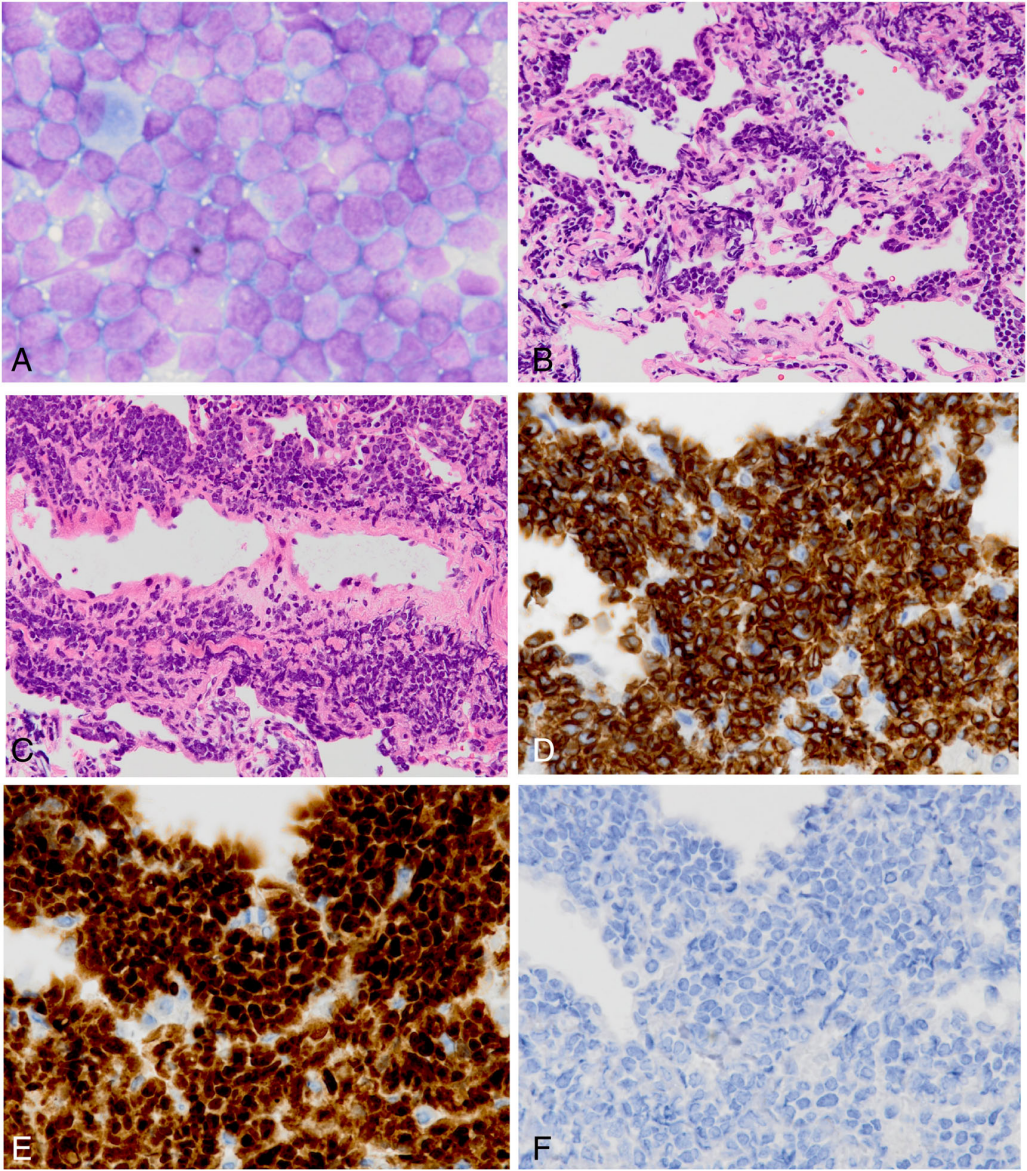
Bronchoalveolar lavage fluid taken from B5 mainly consisted of atypical lymphoid cells (A; 600×). Transbronchial lung biopsied specimens from which randomly obtained right lobes demonstrated numerous atypical lymphoid cells invaded in the alveolar spaces and/or alveolar septa (B; 200×) and in the vein wall (C; 200×). Immunohistochemical analysis was positive for CD3 (D; 400×) and terminal deoxynucleotidyl transferase (TdT) (E; 400×) but negative for CD20 (F; 400×).

Bronchoscopy was performed immediately, and bronchoalveolar lavage fluid (BALF) via B5 appeared milky white. Total BALF cell count was 414 × 10^5^ cells/mL; 93% of the cells were atypical lymphoid cells, and 7% were alveolar macrophages (Fig. 2A; 600×). The CD4‐to‐CD8 lymphocyte ratio was 0.04. No bacteria or fungi were cultured, and DNA polymerase chain reaction was negative for *Pneumocystis jirovecii*. The randomly obtained right lung tissue sampled by trans‐bronchial biopsy showed numerous atypical lymphoid cells located in the alveolar spaces/septa (Fig. 2B; 200×) and the walls of the veins (Fig. 2C; 200×). Immunohistochemical staining was positive for CD3 (Fig. 2D; 400×) and terminal deoxynucleotidyl transferase (TdT) (Fig. 2E; 400×) but negative for CD20 (Fig. 2F; 400×). He was thus diagnosed with recurrent T‐ALL/LBL of the lung.

The patient was further treated with an additional hyper CVAD regimen (C: cyclophosphamide 300 mg/m^2^; V: vincristine 2 mg/body weight; A: adriamycin 50 mg/m^2^; and D: dexamethasone 40 mg/body weight), which resulted in resolution of the subcutaneous tumour and abnormal lung shadows. However, T‐ALL/LBL recurred again in the lung two months later, and the patient died two months after that.

## Discussion

ALL/LBL is a clonal haematopoietic stem cell disorder of B‐ or T‐cell origin and accounts for 2–5% of non‐Hodgkin lymphoma cases in adults. Although T‐ALL/LBL comprises 80–90% of all ALL/LBL cases, the prevalence of lung involvement and radiological findings has been unclear, and few cases of T‐ALL/LBL with lung involvement evaluated in the view of pathological‐radiological correlation have been reported to date in the literature. Differential diagnosis for CT findings of pulmonary non‐Hodgkin lymphoma are diverse and include inflammation or infection (i.e. fungal or mycobacteria) of various aetiology. In addition, sarcoidosis, lymphangitic carcinomatosis, and idiopathic interstitial pneumonia should be considered [2]. The present case confirmed that the severe lung involvement emerged as infiltrates of numerous atypical lymphoid cells both in the alveolar spaces and pulmonary interstitium. Importantly, we successfully compared the radiological and pathological findings with respect to the following: (i) GGO corresponds to the infiltration of atypical lymphoid cells in the alveolar space/septa; (ii) thickening of the interlobular septa corresponds to the infiltration of the atypical lymphoid cells into the perivascular area. Presumably, thickening of the BVBs and scattered tiny nodules in both the lower lung lobes indicated the infiltration of the atypical lymphoid cells to the lymphatic vessels or perilymphatic/haematological spread, respectively.

According to previous reports, myeloid leukaemias such as acute myeloid leukaemia and chronic myeloid leukaemia can generate leucostasis accompanied by diffuse alveolar damage if the total white blood cell count is between 100,000 and 500,000/mm^3^, with a predominance of immature forms [1]. Furthermore, the radiological manifestation of pulmonary leukaemic cell infiltration in myeloid leukaemias mainly consists of bilateral thickening of BVBs, interlobular septa, nodules, and consolidation [3, 4]. From this point of view, the present case had neither blast cells nor hyperleucocytosis in the blood; however, lymphoblasts aggressively infiltrated the lung as the main target organ. Taken together, our findings suggest that even if ALL/LBL patients have no hyperleucocytosis and blast cells in the blood, the emergence of unexplained lung infiltrates should raise the possibility of lung involvement, similar to pulmonary involvement in myeloid leukaemias.

### Disclosure Statement

Appropriate written informed consent was obtained for publication of this case report and accompanying images.
